# Validation of Immunohistochemistry for the Detection of *BRAF* V600E-Mutated Lung Adenocarcinomas

**DOI:** 10.3390/cancers11060866

**Published:** 2019-06-21

**Authors:** Chien-Hung Gow, Min-Shu Hsieh, Yen-Ting Lin, Yi-Nan Liu, Jin-Yuan Shih

**Affiliations:** 1 Department of Internal Medicine, Far Eastern Memorial Hospital, New Taipei City 22060, Taiwan; gowchien@gmail.com; 2 Department of Internal Medicine, National Taiwan University Hospital and College of Medicine, National Taiwan University, Taipei 10002, Taiwan; shwansi.formosa@gmail.com (Y.-T.L.); benson1032@gmail.com (Y.-N.L.); 3 Department of Healthcare Information and Management, Ming-Chuan University, Taoyuan 33348, Taiwan; 4 Department of Pathology, National Taiwan University Hospital, Taipei 10002, Taiwan; mshsieh065@gmail.com; 5 Graduate Institute of Clinical Medicine, National Taiwan University, Taipei 10002, Taiwan; 6 Department of Medical Research, National Taiwan University Hospital, Taipei 10002, Taiwan

**Keywords:** BRAF, immunohistochemistry, lung adenocarcinoma, overall survival, pemetrexed

## Abstract

*BRAF* V600E mutation, a missense mutation in exon 15 resulting in valine substitution for glutamate at position 600 within the kinase domain of BRAF oncogene, is found in a subset of lung adenocarcinoma (ADC). The usefulness of immunohistochemistry (IHC) as an alternative diagnostic tool has not been validated. Moreover, the clinical information of patients with *BRAF* V600E-mutated lung ADC is limited. We retrospectively identified 31 lung ADCs diagnosed with *BRAF* V600E mutation by standard molecular sequencing methods and reviewed their clinical characteristics and pathological features. An anti-BRAF V600E monoclonal VE1 antibody for IHC was used to confirm the expression patterns. The series was comprised of 99 cases, 29 with *BRAF* V600E mutation and 70 without *BRAF* V600E but with other types or undetected mutations. The majority of *BRAF* V600E-mutated biopsied tissues were poorly differentiated and micropapillary patterns. Application of the IHC VE1 assay was highly feasible in primary/metastatic sites or effusion blocks, yielding positive findings in 28 of 29 (96.6%) *BRAF* V600E-mutated tumors and negative results in 69 of 70 (98.6%) tumors harboring other types or undetected mutations. Patients who received pemetrexed/platinum-based rather than mutation-targeted chemotherapy as the first-line therapy for metastatic disease showed median overall survival of 15.5 months. Our findings indicated that VE1 antibody-based IHC analysis demonstrated high sensitivity and specificity to detect *BRAF* V600E-mutated lung ADCs in tissues from primary or metastatic sites.

## 1. Introduction

In the past decade, individualizing molecular targeted therapy based on specific driver oncogene aberrations has provided a powerful and favorable approach to the treatment of non-small cell lung cancer (NSCLC) [1,2,3]. One of the important driver gene mutations, which leads to signaling pathway dysregulation in lung cancer, occurs in v-raf murine sarcoma viral oncogenes homolog B1 (*BRAF*) [4]. BRAF protein, a member of the RAF (rapidly accelerated fibrosarcoma) family of serine/threonine protein kinases, functions to regulate the mitogen-activated protein kinase (MAPK)/extracellular signal-regulated kinase (ERK) pathway. Extracellular signaling activates RAS, a member of the superfamily of guanine nucleotide triphosphatases (GTPases), and subsequently phosphorylates downstream BRAF. Mutations in the BRAF kinase domain lead to increased BRAF kinase activity and can initiate tumorigenesis [5]. In particular, a thymine to adenine single-base change at position 1799 in exon 15, which results in the V600E mutation, is observed in 1–2% of lung ADC [6]. This mutation has been shown to exhibit higher basal kinase activity than that of the wild-type protein [7], and increase transforming capacity in NIH3T3 cells [8]. A lung cancer mouse model by inducible expression of BRAF V600E in the lung epithelial compartment led to development of lung adenocarcinoma in vivo. Deinduction of BRAF V600E decreased the level of constitutively activated MAPK pathway and led to lung tumor regression [9]. Recently, the U.S. Food and Drug Administration (FDA) approved the combination of dabrafenib and trametinib for the treatment of patients with *BRAF* V600E mutation-positive NSCLC [10]. Currently, the standard diagnostic method approved by the FDA is the next-generation sequencing oncology panel test (Oncomine™ Dx Target Test, Thermo Fisher Scientific Inc. USA), which has been shown to accurately and reliably detect patients with NSCLC carrying the *BRAF* V600E mutation [10].

Nevertheless, the best treatment strategies may not always be feasible in real-world practice because of the increased expense of diagnostic tools and targeted medications. Alternative diagnostic approaches and other treatments of choice should also be considered. Recently, detection of the BRAF V600E mutation by immunohistochemistry (IHC) using the anti-BRAF V600E monoclonal VE1 antibody was found to be possible in melanoma, thyroid carcinoma, and colorectal cancer [11,12,13,14,15]. In NSCLC, only few previous studies that compared the sensitivity and specificity of clone VE1 in detecting BRAF V600E mutation with those of other molecular methodologies [16,17]. Therefore, more studies need to be conducted to elucidate whether IHC with the VE1 antibody might be suitable as an alternative method to detect BRAF V600E mutation in patient with lung ADC. Moreover, the clinical characteristics of patients with lung cancer carrying *BRAF* V600E mutation are not consistent across different studies [18,19,20]. In addition, little is known regarding the real-world clinical outcomes of current treatments in patients not receiving BRAF-targeted therapies. Therefore, in this study we conducted a retrospective study of the demographic features of East Asian patients with lung ADC, validated the diagnostic value of IHC for the detection of the *BRAF* V600E mutation, and analyzed the clinical outcomes.

## 2. Results

### 2.1. Clinical and Pathological Characteristics of BRAF V600E Mutation-Positive Patients with Lung ADC

The clinical characteristics of 31 patients harboring primary *BRAF* V600E mutation and 700 patients with non-*BRAF* V600E mutations (without *BRAF* V600E but with other types or undetected mutations) in East Asian lung ADCs are listed in Table 1. The non-*BRAF* V600E cases included 206 *EGFR* mutations, 56 *ALK* fusions, 26 *KRAS* mutations, 27 *MET* exon 14 deletion *(MET^Δ14^)* mutations, 16 *HER2* mutations, 17 *ROS1* fusions, 4 *RET* fusions, 8 *BRAF* non-V600E mutations, and 340 undetected mutations. For lung ADC patients with *BRAF* V600E, the median age was 67.0 (33 to 87) years; 52% (16 of 31) were men; 39% (12 of 31) were current/former smokers, averaging 40 ± 26 pack-years; and 77 % (24 of 31) had Stage IV NSCLC at initial diagnosis. None of the patients were identified as having concurrent *BRAF* V600E and *EGFR*, *KRAS*, *ALK*, *HER2*, *MET^Δ14^*, *ROS1*, or *RET* gene alterations. Patients harboring *BRAF* V600E mutation tended to have poor Eastern Cooperative Oncology Group (ECOG) performance score (*p* = 0.024). There was no significant difference in age, gender, smoking status, stages at initial diagnosis, and numbers of metastatic sites between *BRAF* V600E mutated and non-mutated cases. 

A total of 29 patients with lung ADC had available tumor samples to evaluate their pathological features. These tissues included surgically resected primary tumors (*n* = 7), bronchial or ultrasonography-guided biopsied primary tumors (*n* = 6), biopsies for metastatic sites (extrapulmonary lymph nodes, *n* = 5; bone metastasis, *n* = 1), and cell blocks created from malignant pleural effusion (*n* = 10). Among the 13 primary tumors diagnosed by surgically resected or biopsy specimens, micropapillary-predominant pattern was the most commonly observed morphologic type (9/13, 69%), followed by acinar-predominant (2/13), papillary-predominant (1/13), and solid-predominant pattern (1/13) (Table 2). For non-primary tumors with *BRAF* V600E mutation, the majority of the metastatic lymph nodes and all malignant pleural effusion blocks exhibited a micropapillary-predominant pattern.

### 2.2. Sensitivity of BRAF V600E (VE1) IHC: Results from 29 BRAF V600-Mutated Lung Cancers

**Figure 1:**
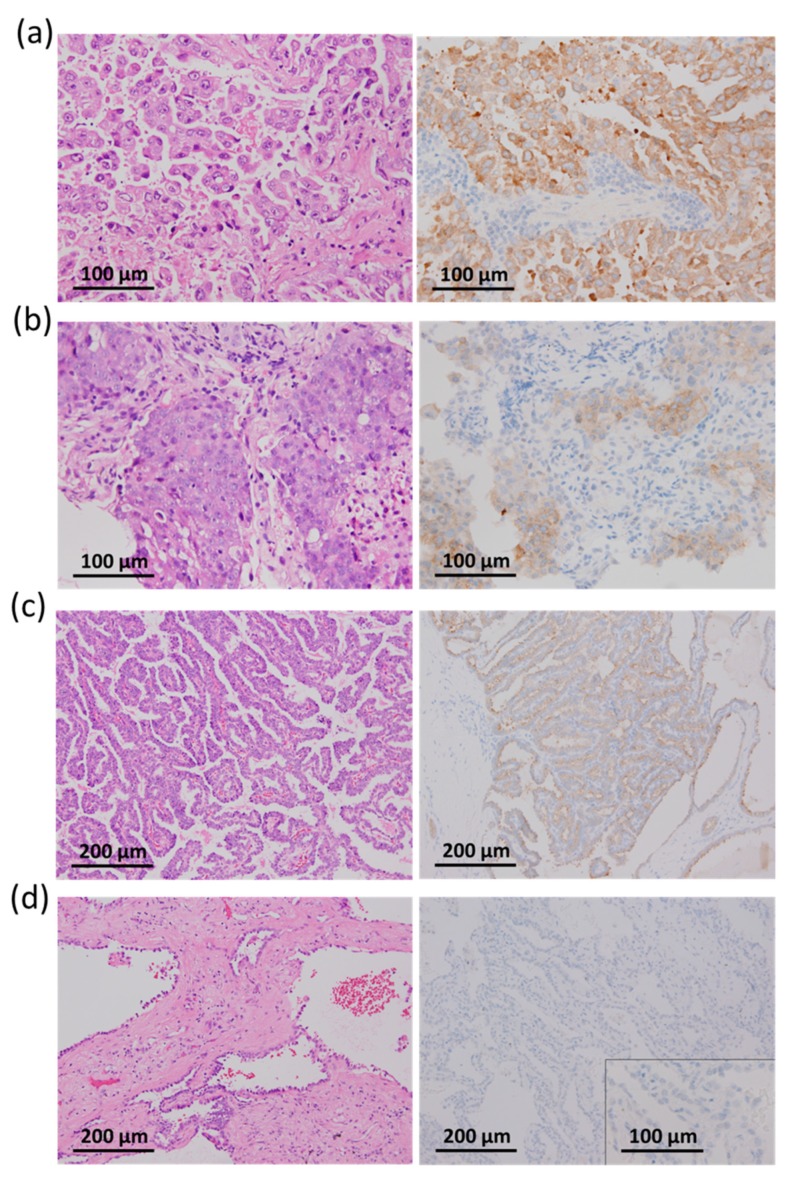
Pathological examination of lung adenocarcinoma with hematoxylin-eosin staining (left panels) and VE1 immunohistochemical staining (right panels). (**a**) A poorly differentiated (grade 3) micropapillary adenocarcinoma with strong cytoplasmic staining. (**b**) A poorly differentiated (grade 3) solid adenocarcinoma with moderate cytoplasmic staining. (**c**). A moderately differentiated (grade 2) papillary adenocarcinoma with weak cytoplasmic staining. (**d**). A moderately differentiated (grade 2), acinar adenocarcinoma carrying wild-type *BRAF* and no detected major driver gene alterations.

**Figure 2:**
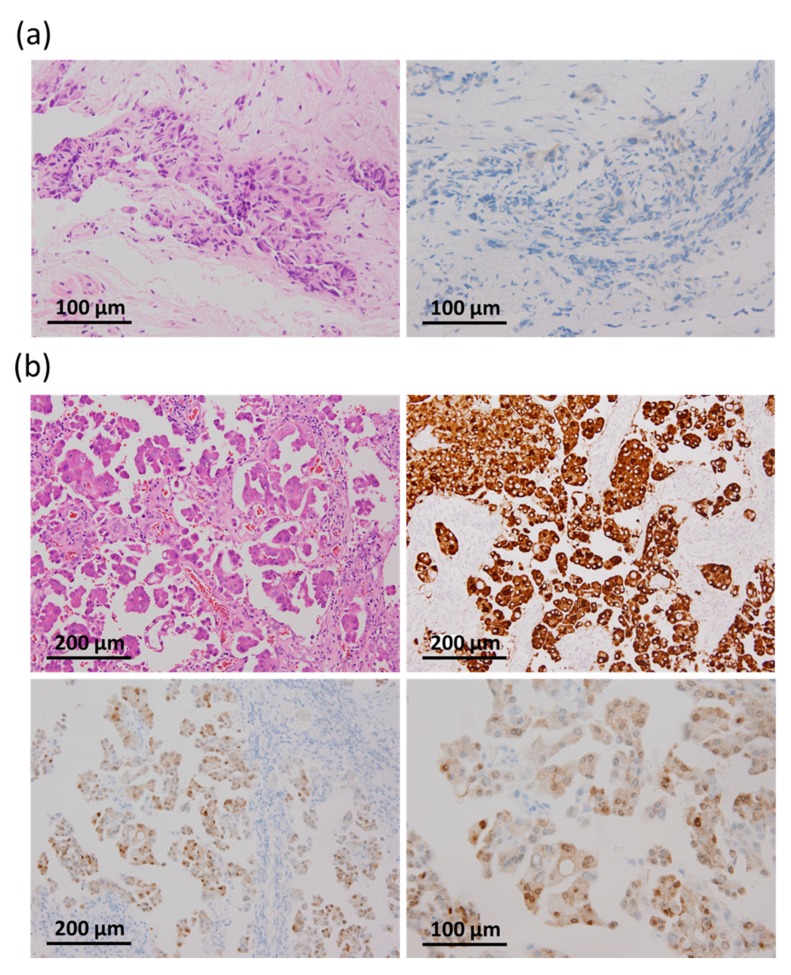
Disconcordant results of BRAF VE1 immunohistochemical (IHC) staining and molecular analysis in two lung adenocarcinoma samples. (**a**). A false negative case. Bronchial biopsy with *BRAF* V600E mutation detected by DNA sequencing shows weak positive cytoplasmic staining in only 10% of tumor cells. Hematoxylin-eosin staining (left panel) and VE1 immunohistochemical staining (right panel) (**b**). A false positive case. The tumor carried an ALK fusion detected by ALK IHC. Upper panels show hematoxylin-eosin staining (left) and ALK IHC (right). Lower panels for VE1 IHC show moderate positive staining in both the nucleus and cytoplasm in 80% of tumor cells (left, 200×; right, 400×).

Using criteria described in the materials and methods, 28 out of 29 lung ADCs (96.6%) with *BRAF* V600E mutation were positive for BRAF V600E (VE1) IHC (Table 3). The IHC staining intensity was weak in 9 cases (1+, 31%), moderate in 9 cases (2+, 31%), and strong in 11 cases (3+, 38%) (Figure 1). The majority of samples exhibited positive cytoplasmic stain in > 80% of tumor cells (22/29, 76%), and 6 cases had positive stain in 60−75% of tumor cells (6/29, 21%). Only one case (case number 8, bronchial biopsy specimen in Table 2) showed weak cytoplasmic stain in only 10% of tumor cells and was considered as negative for the VE1 IHC test in this study (Figure 2a). Overall, based on DNA/RNA sequencing data, our study demonstrated a sensitivity of 96.6% (28/29) for VE1 IHC to detect BRAF V600E mutation in lung ADCs.

### 2.3. Specificity of BRAF V600E (VE1) IHC: Results from 70 Lung Cancers without BRAF V600E Mutations

To determine the specificity of BRAF V600E (VE1) IHC, we randomly selected tumors diagnosed with *BRAF* non-V600E, *EGFR*, *KRAS*, *HER2*, *MET^Δ14^*, *ALK*, *ROS1*, *RET*, or undetected mutations for this study (Table 2). Among 70 cases, only one ALK-fusion-positive lung ADC exhibited both moderate nuclear and cytoplasmic positive staining in 80% of tumor cells (Figure 2b), which was considered as a false positive case. In addition, a case with a ROS1 fusion detected by florescence in situ hybridization analysis showed weak positive cytoplasmic staining in 5% of tumor cells, which was considered as a negative result according to our criteria. Therefore, the specificity of VE1 IHC for tumors without *BRAF* V600E mutation in lung ADCs was 98.6% (69/70).

### 2.4. Clinical Outcomes of BRAF V600E-Mutated Lung ADCs without BRAF-Targeted Therapy

**Figure 3:**
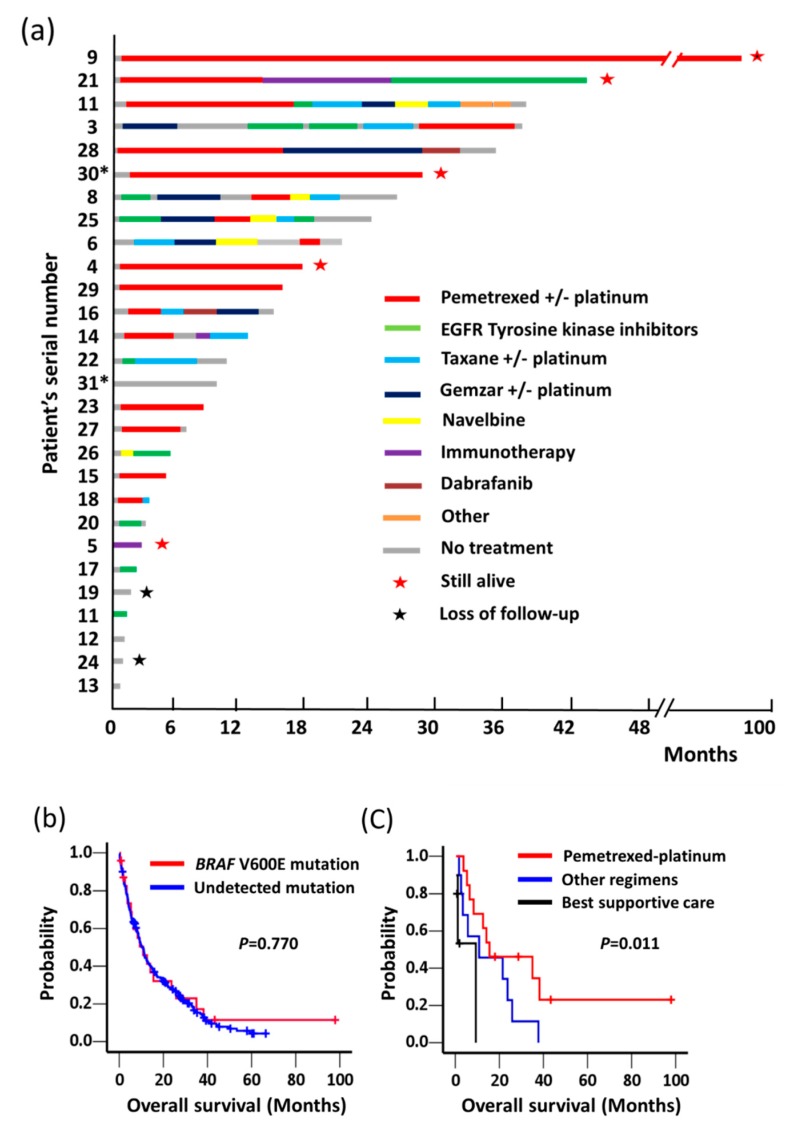
(**a**) The duration of treatments with chemotherapy, targeted and non-targeted therapies, or immunotherapy for patients with metastatic *BRAF* V600E-mutant lung cancers. Symbol with “*” indicates no available tissue sample for BRAF VE1 immunohistochemistry analysis. (**b**) Kaplan–Meier curves of overall survival in patients with lung adenocarcinoma carrying *BRAF* V600E mutation (*n* = 24) and non-*BRAF* V600E with undetected mutation (*n* = 223). (**c**) Kaplan–Meier curves of overall survival in Stage IV *BRAF* V600E-mutated patients who received first-line therapy pemetrexed/platinum then maintenance pemetrexed (*n* = 13), other treatment regimens (*n* = 10), or best supportive care (*n* = 5). *p*-values were calculated using a log-rank test. Symbols with “+” on the plot indicate censored patients.

The detail duration of treatment regimens for patients with metastatic *BRAF* V600E-mutated lung cancers are shown in Figure 3a. Among 31 patients with lung ADC carrying *BRAF* V600E mutation, two patients (Case Numbers 2 and 7 in Table 2) did not relapse and were alive at final assessment. Another patient (Case Number 1 in Table 2) received surgery for a second primary lung cancer. At the data cut-off date, 28 patients had metastatic disease of which 23 received ≥1 line of systemic therapy for metastatic disease (Figure 3a). For the first-line treatments, pemetrexed-platinum doublet chemotherapy was the most common regimen (13 patients, 57% of first-line recipients). Ten patients received other treatment regimens, including EGFR tyrosine kinase inhibitors (*n* = 6), gemcitabine/cisplatin chemotherapy (*n* = 1), navelbine/cisplatin regimen (*n* = 1); docetaxel/cisplatin regimen (*n* = 1), and immunotherapy (pembrolizumab, *n* = 1). Five patients received only best supportive care. 

Overall survival (OS) for Stage IV lung ADC at initial diagnosis were analyzed in patients with *BRAF* V600E or with undetected mutation (no driver mutation/fusion detected in *BRAF*, *EGFR*, *KRAS*, *ALK*, *ROS1*, *RET*, *HER2*, or *MET* genes) (Figure 3b). We observed that 24 patients with *BRAF* V600E mutation (median 10.8 months; 95% confidence interval [CI], 4.3–17.3 months) had similar OS, compared to 223 patients with undetected mutations (median 10.8 months; 95% CI, 9.0–12.8; *p* = 0.770). Although dabrafenib plus trametinib comprises the treatment of choice for patients with lung ADC carrying *BRAF* V600E mutation, none of our patients received such therapy because of financial issues. Therefore, we evaluated the clinical outcome of patients in the absence of dabrafenib and trametinib treatment. The median progression-free survival (PFS) for pemetrexed/platinum followed by maintenance pemetrexed as first-line therapy was 12.6 (95% CI, 4.3–20.9) months. Notably, three patients remained under the maintenance pemetrexed of first-line pemetrexed/cisplatin therapy for 97, 27, and 17 months without disease progression at the data cut-off date. In addition, two patients received third-line single agent dabrafenib treatment in a clinical trial with short PFS (2.8 and 3.9 months). Among 28 patients had metastatic disease, we observed that patients receiving pemetrexed plus platinum then maintenance pemetrexed as first-line therapy exhibited an OS of 15.5 months (95% CI, 0–38 months), which tended to associate with a longer OS than those receiving other regimen (median 10.8 months; 95% CI, 0–33 months) or those receiving best supportive care (median 9.3 months; *p* = 0.011) (Figure 3c).

## 3. Discussion

In the present study, we validated the clinical application of IHC using the VE1 antibody to detect BRAF V600E-mutated lung ADCs from primary lung cancer, metastatic tumors, or malignant pleural effusion cell blocks. We also observed that for patients who were unable to receive BRAF-targeted therapy because of financial issues, pemetrexed-containing regimens might be considered as an important treatment of choice for those with metastatic *BRAF* V600E-lung ADCs. 

The VE1 mouse monoclonal antibody used in the present study constitutes an in vitro diagnostic IHC antibody produced against synthetic peptide sequence (GLATEKSRWSG) that represents the positions from amino acids 596 to 606 in BRAF V600E [21]. This mutation-specific antibody is intended for qualitative staining in sections of formalin-fixed, paraffin-embedded tissue and exhibits a cytoplasmic staining pattern consistent with the cytoplasmic localization of the mutated protein in tumors. This antibody was able to differentiate V600E mutation in the BRAF protein from the wild-type along with other BRAF-mutated proteins [21]. For clinical application, numerous studies have demonstrated that IHC with the VE1 antibody achieved high concordance rates with molecular methods in different types of cancer. The sensitivity and specificity ranged from 85 to 100% and 93 to 100% in melanoma; 89 to 100% and 61.5 to 100% in papillary thyroid carcinomas, and 71 to 100% and 68 to 100% in colorectal carcinomas [11]. Other malignancies, such as primary central nervous system neoplasms [22,23], hairy cell leukemia [24], ameloblastoma [25], and ovarian carcinoma [26] all showed robust concordance with molecular methods of detection as well [11]. Clinically, the staining in BRAF V600E-mutant positive cases can be weak, moderate, to strong, and the distribution can be uniform, nearly uniform, or heterogeneous in colorectal carcinoma [15]. 

In lung cancer, to the best of our knowledge, only two studies provided in-depth evaluation of the clinical feasibility and diagnostic value of IHC with the VE1 antibody for the detection of the BRAF V600E mutation (Table 4). Sasaki et al. [16] applied a Dako EnVisionTMFLEX detection system with the VE1 antibody and compared these findings with the results of direct sequencing in Japanese lung ADC, demonstrating that the autostainer IHC VE1 assay exhibited excellent sensitivity (100%) in 5 of 5 *BRAF* V600E-mutated tumors and specificity (95.2%) in 20 of 21 *BRAF* non-V600E tumors. In a *BRAF* non-600E mutation cases, a 3-bp insertion in *BRAF* resulting in the duplication of threonine 599 (599insT) showed positive in the IHC VE1 assay. Another study by Ilie et al. [17] using the VE1 antibody and an automated single-staining system from Ventana Medical Systems demonstrated sensitivity reaching 90% (19 of 21) in *BRAF* V600E-mutated tumors and perfect specificity (19 of 19, 100%) in all *BRAF* non-V600E-mutated tumors. 

The interpretation criteria of BRAF V600E (VE1) IHC in lung cancer have not been established and different criteria were used by different study groups. The Japanese study group (Sasaki, et al.) used a cut-off value of at least 50% of tumor cells with positive staining irrespective of staining intensity [16]. Ilie et al. used a different method and only cases with a distinct, strong and homogenous signal observed in the cytoplasm of all carcinoma cells were considered positive [17]. The criteria proposed by the manufacturer (Ventana Medical Systems) is positive signal when cells that exhibit unequivocal cytoplasmic staining and the intensity may range from weak to moderate, while negative signal is characterized by an absence of any detectable signal. Our data showed that VE1 IHC in lung cancer is similar to colorectal cancer as the staining intensity may be weak to strong and most positive cases had a near-uniform staining pattern. Therefore, in this study we followed the VE1 IHC interpretation criteria used by the Japanese study group (Sasaki, et al.) and IHC was considered as positive when there was positive cytoplasmic staining in ≥50% of tumor cells. 

To date, however, no study specifically had examined whether VE1 IHC would cross-react with other driver mutations in lung ADCs. Among all 70 non-*BRAF* V600E-mutated cases, only one case with ALK fusion (confirmed by positive ALK[D5F3] IHC) provided a false positive result with positive cytoplasmic and nuclear staining in 80% of tumor cells. This sample was validated as carrying the wild-type *BRAF* gene by direct DNA sequencing; moreover, clinical data showed partial response to an ALK inhibitor (crizotinib) with a progression-free survival (PSF) of 7 months. Among 29 BRAF V600E-mutated cases, only one case (using a bronchial biopsy specimen) yielded a false negative result, which would be associated with inadequate tumor samples. Therefore, we suggest that under certain conditions, such as small tissue samples with a limited number of tumor cells, the BRAF V600E (VE1) IHC test should be more carefully evaluated and rebiopsy for molecular testing should be considered. Nevertheless, together with previous reports, we found that the BRAF V600E (VE1) IHC test was a highly sensitive (94.5%) and specific (98.2) method to detect BRAF V600E mutations in lung ADCs (Table 4).

As previously reported from European studies, *BRAF* V600E mutations were more prevalent in females, more likely to arise in light/never-smokers, and associated with a median age of 67 to 69 years [18,27,28]. The present study also demonstrated a similar age distribution in pure Asian patients. However, our data showed slight mutation prevalence in East Asian men. On histologic pattern analysis, a previous study of lung ADCs demonstrated that the micropapillary pattern-positive patients with stage IA ADC exhibited worse 5- and 10-year OS rates compared to those of micropapillary pattern-negative patients, indicating micropapillary pattern as being an aggressive tumor feature [29]. In the present study, we observed that the majority of *BRAF* V600E-mutated lung ADCs presented a micropapillary-predominant pattern, either in primary lung tumors or metastatic sites, which was consistent with other reports [17,27,30]. Therefore, patients with *BRAF* V600E mutated lung ADCs might have shorter survival than those with non-V600E mutations that were non-micropaillary patterns in majority of tumors, which shown in previous studies [17,31].

Recently, a phase II trial demonstrated that the combination of dabrafenib and trametinib as the first-line treatment showed clinically meaningful antitumor activity and a manageable safety profile in patients with previously untreated *BRAF* V600E-mutated metastatic NSCLC [10,32]. Therefore, the concurrent dabrafenib and trametinib administration is currently considered as an optimal treatment in *BRAF* V600E-mutant metastatic NSCLC. However, despite clinical benefit, targeted therapies may be associated with issues of unaffordability and increase financial burden in real-world cancer management [33]. In the present study, we observed that patients who received pemetrexed/platinum-based chemotherapy followed by pemetrexed continuation maintenance therapy as the first-line treatment for metastatic disease rather than targeted therapy demonstrated a PFS of 12.6 months and a median OS of 15.5 months. The median OS in this study was comparable to that obtained by patients with advanced nonsquamous NSCLC in the phase III PARAMOUNT trial (13.9 months) [34]. Recently, similar median OS data were reported in studies from Tissot et al. (16 months) [35] and Ding et al. (14.7 months) [36] in Stage IV *BRAF* V600E-mutant metastatic NSCLC. Other studies demonstrated to a better median OS, which might be attributed to the benefit to targeted therapy with BRAF/MEK inhibitors [37,38]. Nevertheless, in our study, we reported that a subset of patients with *BRAF* V600E-mutant metastatic NSCLC may obtain clinical benefit from pemetrexed-based chemotherapy. In particular, a 49-year-old man with initial Stage IV lung ADC who received pemetrexed/cisplatin followed by pemetrexed monotherapy for eight years exhibited favorable outcome and is still under pemetrexed treatment. A similar patient with Stage IIIB NSCLC has also been reported recently, who showed no disease progression for over eight years under pemetrexed treatment [39]. 

Some potential limitations of the present study include the retrospective design of the study and the relatively small sample size. In addition, although the treatment modalities in our institutions were standardized, they may have varied as they depended on the individual decision of each physician or the personal choice of each patient. Nevertheless, this study provides important insight on diagnostic method and a potential treatment of choice for lung ADC patients harboring *BRAF* V600E mutation. Further large scale, multi-center, standardized, and prospective studies are needed to clarify these findings.

## 4. Materials and Methods 

### 4.1. Study Design

We retrospectively reviewed medical record of primary *BRAF* V600E-mutated lung ADC at the National Taiwan University Hospital (Taipei, Taiwan). In total, 31 lung cancer specimens with *BRAF* V600E mutation diagnosed by direct DNA sequencing or reverse transcription-polymerase chain reaction (RT-PCR) were identified. The clinical data of 700 non-*BRAF* V600E lung ADC patients were retrieved from our previous published cohort study to compare with *BRAF* V600E-mutated patients [40]. The clinicopathologic characteristics of the 731 patients were recorded including age, gender, smoking status, histological subtype, initial stage [41], Eastern Cooperative Oncology Group performance status [42], initial metastatic sites, and chemotherapy/targeted therapy. Progression-free survival was defined as the time of treatment initiation and tumor progression or death from any cause, with censoring of patients who were lost to follow-up. Overall survival was defined as the period from the date of initial diagnosis of Stage IV lung cancer to the date of death (or censored at the date of last follow-up/loss of contact). 

For BRAF V600E IHC analyses, we retrieved tumor tissues diagnosed as *BRAF* V600E mutation-positive and without *BRAF* V600E, and used the VE1 clone for the IHC assay. Histologic patterns were recorded. Two patients with inadequate tissue specimens were excluded. Finally, 29 patients with lung cancer (93.5%) were included in the VE1 IHC analysis. The 70 lung tumors with mutations other than *BRAF* V600E or undetected mutations were randomly selected from our 700 cohort patients to determine the specificity of the VE1 IHC assay; these included tumors with *BRAF* non-V600E mutations, major driver mutations (*EGFR*, *KRAS*, *HER2*, *MET^Δ14^*, *ALK*, *ROS1*, and *RET*), and no-detectable mutations. The study was approved by the Institutional Review Board of the National Taiwan University Hospital (NTUH-Research Ethics Committee number: 201807075RINC; October 1, 2018).

### 4.2. Mutation Analyses

Molecular analyses for *BRAF* and other major driver alterations were performed. The specimen preparation, DNA/RNA extraction, RT-PCR, and sequencing of *EGFR*, *KRAS*, *HER2*, and *MET^Δ14^* mutations, along with *ALK*, *RET*, and *ROS1* fusions were performed individually as described previously [40,43,44,45]. A portion of ALK fusions was determined by IHC based on standard protocol [46]. A portion of ROS1 fusions was determined by fluorescence in situ hybridization analyses as previous described [45]. To validate detected *BRAF* V600E mutations, RT-PCR was performed on RNA samples based on the manufacturer’s protocol and as previously described [43]. The primers used were: forward 5′-TCCAGGACCTCAGCGAGAAAGGA-3′) and reverse (5′-TGATGACTTCTGGTGCCATCCACA-3′). For *BRAF* V600E genomic DNA sequencing, the template consisted of 100 ng of genomic DNA. PCR reactions were performed using standard PCR conditions [95 °C × 3 min; 95 °C × 30 s, 60 °C × 30 s, 72 °C × 1 min, for 35 cycles; then 72 °C × 10 min, 50 µL reactions] with forward (5′-TGTTTTCCTTTACTTACTACACCTCA-3′) and reverse (5′-CCACAAAATGGATCCAGACA-3′) primers. The resulting products were directly sequenced in both directions using the same primers as RT-PCR or DNA sequencing mentioned above. National Center for Biotechnology Information *BRAF* (NM_004333.5) was used as the reference sequence.

### 4.3. Immunohistochemistry 

Tissue sections (4 μm thick) were dewaxed and rehydrated. For hematoxylin-eosin staining, sections were reacted with hemalum followed by counterstaining with eosin. For IHC staining, sections were deparaffinized using EZ Prep™ solution (Ventana Medical Systems, Inc., Tucson, AZ, USA) and subjected to a 64 min pretreatment using Cell Condition 1 solution (Ventana Medical Systems). The slides were incubated with VE1 antibody (Ventana Medical Systems) for 16 min. The Optiview DAB Detection Kit (Ventana Medical Systems) was used to detect protein expression. All sections were counterstained with hematoxylin. The IHC was performed on the Benchmark XT platform (Ventana Medical Systems). The staining intensity was recorded as 0 (negative), 1+ (weak cytoplasmic staining), 2+ (moderate cytoplasmic staining), and 3+ (strong cytoplasmic staining). The percentage of tumor cells with positive staining was recorded semiquantitatively in each case. Nuclear staining was not considered as positive for BRAF V600E according to manufacturer instruction. BRAF V600E (VE1) IHC was considered as positive when there was positive cytoplasmic staining in ≥50% of tumor cells.

### 4.4. Statistical Analyses 

For the clinicopathological characteristics of the patients, all categorical variables were analyzed using a chi-square test. PFS and OS curves were plotted using the Kaplan–Meier method. All statistical analyses were performed using Statistical Package for the Social Sciences software for Windows, version 17.0 (SPSS Inc., Chicago, IL, USA).

## 5. Conclusions

We observed that using the VE1 antibody for IHC analysis afforded highly sensitivity and specificity to detect *BRAF* V600E-mutated lung ADCs in tissues from primary lung cancer, metastatic tumors, or malignant pleural effusion cell blocks. Conventional chemotherapy, particularly pemetrexed/platinum-based first-line therapy, may be considered as an important treatment of choice for patients unable to receive the combination of dabrafenib and trametinib yet harboring metastatic *BRAF* V600E-mutated lung ADC.

## Figures and Tables

**Table 1:** Clinical characteristics of patients with lung adenocarcinoma with *BRAF* V600E mutation (*n* = 31) or non-*BRAF* V600E mutations (*n* = 700).

Clinical Characteristic	*BRAF* V600E	Non*-BRAF* V600E	*p*-Value ^#^
Patients, *n*	31	700	
Age, years			
Median (range)	67.0 (33–87)	65.7 (27–93)	0.455
>70, *n* (%)	14 (45)	268 (38)	
Gender, *n* (%)			
M	16 (52)	381 (54)	
F	15 (48)	319 (46)	0.854
Smokers, *n* (%)	12 (39)	267 (38)	
Pack-years, average/SD	40/26	N/A	1.000
ECOG PS, *n* (%)			
0−1	21 (68)	589 (84)	
2−4	10 (32)	111 (16)	0.024 *
Stage, *n* (%)			
I−IIIB	7 (23)	228 (33)	
IV	24 (77)	472 (67)	0.245
Metastatic sites			
0−1	14 (58)	255 (54)	
≥2	10 (42)	217 (46)	0.834

Abbreviations: ECOG, Eastern Cooperative Oncology Group; F, female; M, male; N, number; N/A, not available; PS, performance status; SD, standard deviation. ^#^ *p*-values were calculated using a two-sided chi-square test. * Indicates values that are statistically significant (*p* < 0.05).

**Table 2:** Pathological characteristics of patients with lung adenocarcinoma (*n* = 29) harboring *BRAF* V600E mutation.

Patient’s Serial No.	Age, Years	Sex	Sample	Stage	Histological Pattern	Grade ^a^	IHC Score ^b^	IHC Positive Percentage
1	61	F	SUR	IIIA	Acinar	2	2+	100%
2	80	F	SUR	IB	Acinar	2	1+	70%
3	66	M	SUR	IIIA	Micropapillary	3	1+	80%
4	61	F	SUR	IIIA	Micropapillary	3	2+	70%
5	54	F	SUR	IIIA	Micropapillary	3	3+	100%
6	71	M	SUR	IIIB	Micropapillary	3	3+	100%
7	59	F	SUR	IB	Papillary	2	1+	60%
8	67	M	Bron B	IV	Micropapillary	3	1+	10%
9	49	M	Bron B	IV	Micropapillary	3	2+	70%
10	67	M	Bron B	IV	Micropapillary	3	2+	75%
11	67	M	Echo B	IV	Micropapillary	3	2+	75%
12	55	M	Echo B	IV	Micropapillary	3	3+	90%
13	85	F	Echo B	IV	Solid	3	2+	95%
14	65	F	LN	IV	Micropapillary	3	1+	100%
15	67	M	LN	IV	Micropapillary	3	2+	95%
16	71	F	LN	IV	Micropapillary	3	3+	100%
17	87	F	LN	IV	Solid	3	3+	95%
18	65	F	LN	IV	Solid	3	3+	100%
19	50	M	Bone	IV	Acinar	2	3+	90%
20	69	F	MPE	IV	Micropapillary	N/A	1+	75%
21	78	F	MPE	IV	Micropapillary	N/A	2+	90%
22	74	F	MPE	IV	Micropapillary	N/A	1+	80%
23	85	M	MPE	IV	Micropapillary	N/A	1+	90%
24	85	M	MPE	IV	Micropapillary	N/A	2+	80%
25	40	M	MPE	IV	Micropapillary	N/A	2+	95%
26	78	F	MPE	IV	Micropapillary	N/A	3+	90%
27	78	M	MPE	IV	Micropapillary	N/A	3+	95%
28	78	M	MPE	IV	Micropapillary	N/A	3+	100%
29	67	M	MPE	IV	Micropapillary	N/A	3+	100%

Abbreviations: Bron B, bronchoscopic biopsy; Echo B, echo-guided biopsy; F, female; IHC, immunohistochemistry; LN, lymph node; M, male; MPE, malignant pleural effusion; N/A, not available; No., number; SUR, surgery. ^a^ Applied only to surgical or biopsy tissues. ^b^ IHC score: 3+, strong cytoplasmic staining; 2+, moderate cytoplasmic staining; 1+, weak cytoplasmic staining; 0, negative.

**Table 3:** BRAF VE1 immunohistochemical staining for *BRAF* V600E, *BRAF* non-V600E, and other major driver mutations in lung adenocarcinoma.

Driver Gene Alteration (Case Numbers)	Gene Alteration (Numbers)	Mutation Detection Methods	BRAF (VE1) IHC Positive * (Numbers)	Positive %
*BRAF* V600E (29)	V600E (29)	Sequencing	28 *^a^*	97
*BRAF* non-V600E (8)	G469A (3) G469V (1) K483E (1) D594G (2) L597R (1)	Sequencing	0	0
*EGFR* (14)	exon 18 G719A (2) exon 19 deletion (4) del L747-A750insP (1) del E746-A750 (2) del E746-S752insV (1) exon 21 L858R (7) exon 21 L861Q (1)	Sequencing	0	0
*KRAS* (9)	G12A (1) G12C (3) G12D (2) G12V (2) G12R (1)	Sequencing	0	0
*ALK* fusion (10)	ALK IHC positive (5) *EML4-ALK* fusion (5)	IHC Sequencing	1*^b^*	10
*ROS1* fusion (5)	ROS1 FISH positive (4) *CD74* exon6-*ROS1* exon34 (1)	FISH Sequencing	0*^c^*	0
*RET* fusion (4)	*KIF5B* exon15-*RET* exon12 (2) *CCDC* exon1-*RET* exon12 (1) *CCDC* exon5-*RET* exon11 (1)	Sequencing	0	0
*HER2* (5)	A775_G776insYVMA (4) P780_Y781insGSP (1)	Sequencing	0	0
*MET* (5)	exon 14 skipping (5)	Sequencing	0	0
Undetected mutation ^#^ (10)	No mutation/fusion detected (10)	Sequencing	0	0

Abbreviations: Del, deletion; FISH, fluorescence in situ hybridization; IHC, immunohistochemistry; Ins, insertion. * BRAF V600E (VE1) IHC was considered as positive when there was positive cytoplasmic staining in ≧50% of tumor cells. ^#^ No driver mutation/fusion detected in *BRAF*, *EGFR*, *KRAS*, *ALK*, *ROS1*, *RET*, *HER2*, or *MET* genes. ^a^ A case with *BRAF* V600E mutation showed IHC score 1+ in 10% of tumor cells. ^b^ A case with *ALK* fusion showed IHC 2+ for both nuclear and cytoplasmic staining in 80% of tumor cells. ^c^ A case with *ROS1* fusion showed IHC 1+ in 5% of tumor cells.

**Table 4:** Sensitivity and specificity of immunohistochemical detection for patients with lung adenocarcinoma harboring BRAF V600E in different studies.

Study	Sensitivity	Specificity	Cases for IHC	Assay
Sasaki et al. [16]	5/5 (100%)	20/21 (95.2%)	*BRAF* V600E *BRAF* non-V600E mutations	VE1 Ab, Dako EnVision^TM^ ELEX detection system
Ilie et al. [17]	19/21 (90.5%)	19/19 (100%)	*BRAF* V600E *BRAF* non-V600E mutations	VE1 Ab, Ventana Medical Systems
This study	28/29 (96.6%)	69/70 (98.6%)	*BRAF* V600E *BRAF* non-V600E mutations *EGFR*, *KRAS*, *HER2*, *MET^Δ14^*, *ALK*, *ROS1*, or *RET* mutations Undetected mutation ^#^	VE1 Ab, Ventana Medical Systems
Total	52/55 (94.5%)	108/110 (98.2%)		

Abbreviations: Ref., reference; IHC, immunohistochemistry. # No driver mutation/fusion detected in *BRAF*, *EGFR*, *KRAS*, *ALK*, *ROS1*, *RET*, *HER2*, or *MET* genes.
